# Electron microscopy images and morphometric data of SARS-CoV-2 variants in ultrathin plastic sections

**DOI:** 10.1038/s41597-024-04182-3

**Published:** 2024-12-04

**Authors:** Tobias Hoffmann, Janine Michel, Andreas Nitsche, Christin Mache, Jessica Schulze, Thorsten Wolff, Michael Laue

**Affiliations:** 1 Advanced Light and Electron Microscopy, Centre for Biological Threats and Special Pathogens 4 (ZBS 4), Robert Koch Institute, Berlin, Germany; 2 Highly Pathogenic Viruses, Centre for Biological Threats and Special Pathogens 1 (ZBS 1), Robert Koch Institute, Berlin, Germany; 3 Influenza and Other Respiratory Viruses (Unit 17), Robert Koch Institute, Berlin, Germany

**Keywords:** SARS-CoV-2, Viral infection

## Abstract

Conventional thin section electron microscopy of viral pathogens, such as the pandemic SARS-CoV-2, can provide structural information on the virus particle phenotype and its evolution. We recorded about 900 transmission electron microscopy images of different SARS-CoV-2 variants, including Alpha (B.1.1.7), Beta (B.1.351), Delta (B.1.617.2) and Omicron BA.2 (B.1.1.529) and determined various morphometric parameters, such as maximal diameter and spike number, using a previously published measurement method. The datasets of the evolved virus variants were supplemented with images and measurements of the early SARS-CoV-2 isolates Munich929 and Italy-INMI1 to allow direct comparison. Infected Vero cell cultures were cultivated under comparable conditions to produce the viruses for imaging and morphometric analysis. The images and measurements can be used as a basis to analyse the morphometric changes of further evolving viruses at the particle level or for developing automated image processing workflows and analysis.

## Background & Summary

In the Wuhan region of China, a novel type of lung disease emerged for the first time at the end of 2019. Initially designated as 2019-nCoV, the virus was subsequently renamed SARS-CoV-2 (Severe acute respiratory syndrome coronavirus type 2). The novel coronavirus is the cause of COVID-19, a disease that can lead to organ failure and death^1^. Coronaviruses are widespread in the animal kingdom and likely expand their host range due to their ability of homologous recombination^2^. Betacoronaviruses, such as SARS-CoV-2 and its relatives, SARS-CoV (79% genetic sequence identity, now grouped in the same species *Betacoronavirus pandemicus*) and MERS-CoV, pose a significant threat for humans and appeared within the last 20 years which underlines the large potential of the Coronaviruses to adapt to new hosts.

**Fig. 1:**
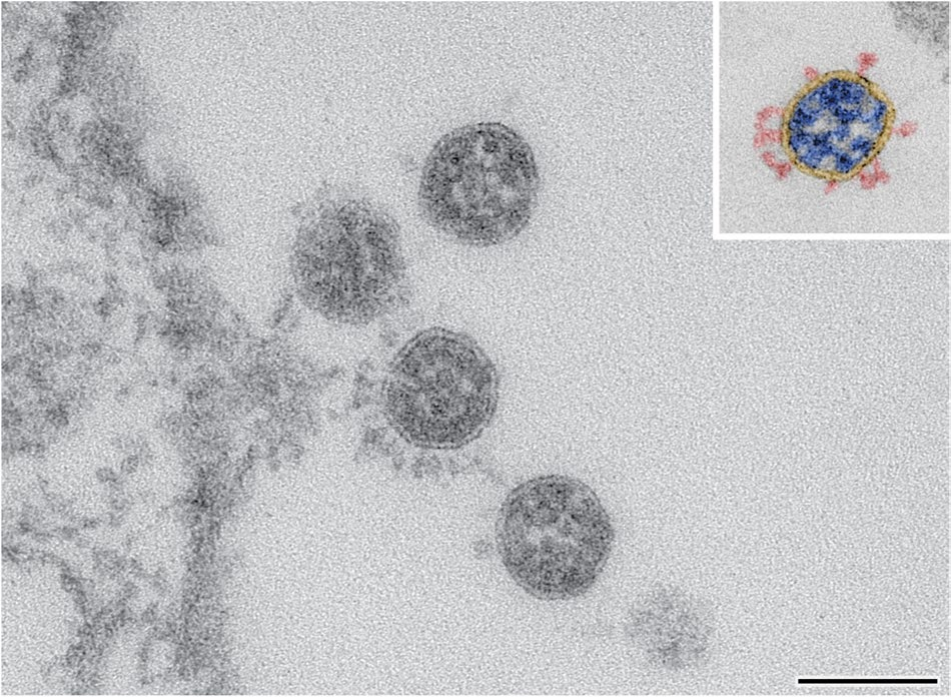
Example of an image of four SARS-CoV-2 particles from dataset 04 (Beta, B.1.351) recorded by transmission electron microscopy at a magnification of 135,000x. The four virus particles are localized in close proximity to the Vero cell surface visible on the left side. The inset shows a colored particle profile to illustrate the spikes (red), the virus-enveloping membrane (yellow) and the ribonucleoprotein (blue) of the virus. Scale bar = 100 nm.

SARS-CoV-2 caused a global pandemic and has acquired significant genome changes since its first emergence. Coronaviruses are membrane-enveloped RNA viruses with a diameter of 80 to 140 nm^3^. The large surface (S) protein forms so-called spike trimers with a length of 20 to 25 nm (Fig. 1)^3^. The single-stranded RNA genome with positive polarity has a size of approximately 30 kilobases. It codes for the four structural proteins S, E, M, and N, as well as other non-structural proteins responsible for RNA replication. The S, E, and M proteins are embedded in the virus membrane. The virus membrane envelops the nucleocapsid, which is composed of the RNA genome and the N protein (ribonucleoprotein)^4^ (Fig. 1). The S protein is responsible for the entry into the host cell. Each S monomer consists of a S1 subunit with the receptor binding domain (RBD) and a S2 subunit, which is responsible for the fusion process with the host cell membrane. To enter the host cell, SARS-CoV-2 mainly uses the cellular ACE-2 receptor and this process is supported by the cellular protease TMPRSS2^5^. However, a possible shift towards the alternative endosomal entry route of the virus^5^ is discussed for the omicron variant^6,7^.

The essential role of the S protein and the surface spikes for cellular infection of the virus^5^ was the reason for its extensive molecular and structural investigation^8^. Besides variations in the protein structure at the molecular level, which define the affinity and specificity for the cellular receptor, density of the S protein and spikes presented at the virus particle surface may have an effect on the infection likelihood and dynamics^9^. Particle size and shape are also considered as important factors for the transmission efficiency and therefore infectivity of viruses that are mainly transmitted by aerosols^10^. Thus, the measurement of spike density, virus particle shape and size are important for understanding virus infectivity and epidemiology.

Transmission electron microscopy (TEM) is a widely utilized technique in virology, as viruses are too small to be examined with a light microscope^11^. The molecular structure of the spike protein has already been fully elucidated using cryo-electron microscopy (cryo-EM) or cryo-electron tomography (cryo-ET)^12^. In the early stages of the pandemic, morphometric data of SARS-CoV-2 isolates^13–15^ and of intracellular virus particles^16^ obtained by cryo-ET have been published. Although cryo-EM and cryo-ET are the optimal methods for investigating the ultrastructure and structural biology of viruses, conventional EM using ultra-thin plastic sections remain invaluable. In particular, complex multicellular objects or pathological material from patients still can be more effectively examined using conventional EM than by cryo-EM or cryo-ET, because the investigations usually require extensive searching and screening to find regions of interest and/or target structures which still is a limit in cryo-EM or cryo-ET due to dose and sample size constraints. Moreover, the throughput of cryo-EM and especially of cryo-ET, in terms of sample number, still is limited, especially if the structures of interest are present at sub-optimal concentration. To obtain morphometric reference data of SARS-CoV-2 particles for conventional EM, we developed and validated a workflow in a previous study using measurements of virus profiles in thin plastic sections and compared the results with measurements obtained from entire virus particles^3^. The results produced with this comparatively simple approach provided a reasonable estimator for the spike density of entire virus particles which were very similar to results obtained by cryo-ET^3^. As a consequence, we started producing samples and images of SARS-CoV-2 variants that appeared during the ongoing pandemic and monitored continuously their morphological characteristics by using measurements of virus particle profiles as estimators for the values of entire virus particles. This data publication describes some of the data which we have collected as a follow-up to our previous paper which provided data on the comparison between an early isolate of SARS-CoV-2 and an isolate of SARS-CoV^3^.

The WHO had previously defined five so-called “Variants of Concern” (VOCs) during the pandemic, which had altered pathogen properties such as increased transmissibility, virulence, or immune escape^17^. In the present study, four of these five VOCs were examined using the thin plastic section technique^3^ for transmission electron microscopy: Alpha (B.1.1.7)^18^, Beta (B.1.351)^19^, Delta (B.1.617.2)^20^ and Omicron BA.2 (B.1.1.529)^21^. In addition to the VOCs, two reference isolates from the beginning of the pandemic, Munich929^22,23^ and Italy-INMI1^24,25^, were included in the study to allow direct comparison. Table 1 lists the SARS-CoV-2 virus strains analysed in the present study with their main morphological characteristics (maximum particle diameter, particle circumference, spike number/density). The morphometric values of the different virus isolates differ not much with largely overlapping distribution of values. However, a slight tendency is visible. The more dominant SARS-CoV-2 variants Alpha (B.1.1.7)^18^, Delta (B.1.617.2)^20^, and Omicron BA.2 (B.1.1.529)^21,26^ showed a slightly increased spike density compared to the reference strains SARS-CoV-2 Munich929^23^ and Italy-INMI1^25^ which is mainly due to a smaller virus particle size. The less dominant variant SARS-CoV-2 Beta (B.1.351)^19,26^ exhibited a reduced spike density compared to the reference strains Munich929^23^ and Italy-INMI1^25^, with a reduction in the number of spikes per virus profile and a larger particle size. Remarkably, a similar tendency towards an increased spike density of dominant VOCs compared to the spike density in the variants that emerged early in the pandemic was identified in a cryo-ET study that was recently published^27^. Infection studies with appropriate infection systems, which measure the morphometric parameters of the virus populations used, must be conducted to find out about the relevance of the observed morphometric differences.

**Table 1:** Overview about the investigated SARS-CoV-2 isolates and their morphometric characteristics determined in section profiles of virus particles (median of distribution and interquartile range in brackets).

Data set #	Virus isolate	max. profile diameter [nm]	profile circumference [nm]	spikes per virus 2D section profile	spike density per 100 nm circumference
1	SARS-CoV-2 Munich929	95 (10)	285 (27)	9 (6)	2.8
2	SARS-CoV-2 Italy-INMI1	95 (4)	290 (15)	9 (5)	3.2
3	SARS-CoV-2 Alpha (B.1.1.7)	93 (6)	284 (14)	10 (4)	3.5
4	SARS-CoV-2 Beta (B.1.351)	99 (6)	302 (17)	8 (5)	2.6
5	SARS-CoV-2 Delta (B.1.617.2)	92 (6)	281 (17)	10 (4)	3.6
6	SARS-CoV-2 Omicron (B.1.1.529)	93 (5)	280 (15)	10 (5)	3.5

## Methods

### Virus isolates

RNA extraction and semi-quantitative reverse transcription polymerase chain reaction (qRT-PCR) of the variants were performed in accordance with a previously published standard protocol^28^. Virus variants were determined using validated mutation-specific polymerase chain reactions (PCRs) for single nucleotide polymorphism (SNP) N501Y, deletion H69/V70, and PCRs of the TaqMan SARS-CoV-2 mutation panel from Thermo Fisher Scientific, or by sequencing^29^. The following virus isolates were employed for the purposes of electron microscopy measurements:

1. SARS-CoV-2 Munich929^22^ (hCoV-19/Germany/BY-ChVir-929/2020; GISAID: EPI_ISL_406862)
2. SARS-CoV-2 Italy-INMI1^24^ (GenBank: SARS-CoV-2/INMI1-Isolate/2020/Italy: MT066156)
3. SARS-CoV-2 Alpha (B.1.1.7)
4. SARS-CoV-2 Beta (B.1.351)
5. SARS-CoV-2 Delta (B.1.617.2)
6. SARS-CoV-2 Omicron BA.2 (B.1.1.529)

### Cell culture

Vero E6 cells (African green monkey kidney epithelial cell, ECACC, ID: 85020206) were cultured in cell culture flasks with Dulbecco’s Modified Eagle Medium (DMEM) containing 1% L-glutamine and 10% fetal bovine serum for 24 hours at 37 °C and 5% CO2, reaching approximately 70% confluence. To infect the cultures with virus, the medium was removed and a small volume of fresh medium containing a diluted virus stock suspension was added to the cells. Multiplicity of infection (MOI) was approximately MOI 1 for dataset 01 and 0.01 for datasets 02 to 06. After incubation for 30 minutes, fresh medium without virus was added and the cells were further incubated. Cultivation was terminated 24 hours after the addition of the virus suspension by replacing the medium with 2.5% glutaraldehyde in 0.05 M Hepes buffer (pH 7.2) (Datasets 02 to 06) or with 2.5% glutaraldehyde 1% paraformaldehyde in 0.05 M Hepes buffer (pH 7.2) (Dataset 01). Incubation with the fixative lasted at least one hour at room temperature. The fixed cells were then scraped from the culture flasks and collected in centrifuge tubes.

### Sample preparation

Fixed cells were sedimented by centrifugation (3000 *g*, 10 min) using a swing-out rotor and washed twice with 0.05 M Hepes buffer. The cell pellet was heated to 40 °C in a water bath and mixed with 3% low melting point agarose (1:1 [v/v]) at 40 °C. Following a brief incubation for approximately 2-3 minutes at 40 °C, the suspension was centrifuged for 5 minutes at 5000 *g* in a benchtop centrifuge with a fixed-angle rotor and cooled on ice to form a gel. The cell pellet was subsequently excised from the solidified gel block with a razor blade and stored in 2.5% glutaraldehyde in 0.05 M Hepes buffer. Subsequently, the specimens were subjected to postfixation (1% osmium tetroxide in water), *en bloc* contrasting (0.1% tannic acid, 2% uranyl acetate), dehydration, and embedding in epoxy resin^11^. A step-by-step protocol of the entire preparation is provided in the supplementary information of our previous publication^3^.

Ultrathin sections were prepared with an ultramicrotome (UC7, Leica Microsystems, Germany) using a diamond knife (45°, Diatome, Switzerland) at a section thickness setting of 45 nm. The sections were collected on bare copper grids (300 mesh, hexagonal mesh shape), contrasted with 2% uranyl acetate and 0.1% lead citrate, and coated with a thin (2-3 nm) layer of carbon.

### Electron Microscopy (EM)

The ultrathin sections were examined using a transmission-electron microscope (Tecnai Spirit, Thermo Fisher Scientific) equipped with a LaB_6_ filament and operated at 120 kV. The magnification calibration of the microscope was verified using the MAG*I*CAL calibration reference standard for transmission electron microscopy (Technoorg Linda, Hungary). Images were recorded with a side-mounted CCD camera (Phurona, EMSIS, Germany) at full resolution (4112 × 3008 pixels). The images were partly captured using the “drift correction” function integrated into the Phurona software, which resulted in a slightly variable size of the images (4065 to 4112 pixels x 2960 to 3008 pixels).

Extracellular virus particles in ultrathin sections were selected at random at the microscope and were recorded with the side-mounted camera (at a magnification of 135,000x) if they met the following criteria: (1) the particle was morphologically intact; (2) the particle was not deformed (e.g., by pressing against other structures or by impaired assembly); (3) the particle membrane was visible (at least 90%)^3^. A total of 900 image files grouped into six datasets^18–21,23,25^ (Table 2) were recorded from 2-3 section levels per dataset (level intervals were at least 2 µm) to obtain measurable virus particle profiles of approximately 150 different virus particles for each virus population/dataset.

**Table 2:** List of datasets.

Data set #	Virus isolate	number of files	repository
1	SARS-CoV-2 Munich929	150	10.5281/zenodo.13121516
2	SARS-CoV-2 Italy-INMI1	154	10.5281/zenodo.13136112
3	SARS-CoV-2 Alpha (B.1.1.7)	147	10.5281/zenodo.13136744
4	SARS-CoV-2 Beta (B.1.351)	132	10.5281/zenodo.13136767
5	SARS-CoV-2 Delta (B.1.617.2)	153	10.5281/zenodo.13136809
6	SARS-CoV-2 Omicron (B.1.1.529)	164	10.5281/zenodo.13136320

All image files are in TIF-format (16 bit) and were recorded at a pixel size of 0.1641 nm. The images size is 4065 to 4112 pixels by 2960 to 3008 pixels. Each image file shows profiles of one or more virus particles.

### Measurement of virus particle size and spike number

All morphometric measurements of the virus particle profiles, such as size and spike number, were performed in accordance with a previously validated method^3^. Data were obtained from ultrathin plastic sections of 45 nm thickness, which was the lowest sectioning thickness setting that produced regular sections and which allowed the best visualization of virus membrane and of spikes. Particle size measurements were conducted with Fiji-ImageJ^30^ by selecting the outer circumference of the virus membrane with the “polygonal selection” tool and the measurement setting “fit ellipse”. The maximal and minimal diameter of the fitting ellipse, the circumference, as well as shape descriptors such as aspect ratio and circularity (4π*area/perimeter²) were determined. The number of spikes (including spike fragments if recognized) per virus particle profile was counted manually.

## Data Records

A total of 900 image files are publicly available in the Zenodo repository as 16-bit Tif files. The image files are grouped into six datasets^18–21,23,25^ according to the virus population they belong to (six different cell culture experiments with six different virus isolates). Table 2 provides an overview of the six datasets^18–21,23,25^ indicating virus isolate, number of image files and the unique digital object identifier. The image datasets^18–21,23,25^ are supplemented with a spreadsheet table (XLSX-format) of the morphometric measurements for each image and descriptive statistic values for their distribution.

## Technical Validation

The datasets described in this publication^18–21,23,25^ were generated with the methods described in a previously published morphometry study on SARS-CoV and SARS-CoV-2 Italy-INMI1^3^. In this previous study we validated the results of the measurements obtained at virus particle profiles in ultrathin sections. First, we checked whether a biological and technical replicate provided the same result. The experiments demonstrated that the size distributions and spike numbers for SARS-CoV-2 Italy-INMI1 were almost identical in independent replicates suggesting that both, biological and technical variation, are small. Second, we demonstrated, by comparing measurements from virus particle profiles with measurements from 3D-reconstructed complete virus particles, that determination of spike number and particle size using thin section profiles of the viruses, provides valid estimators for these parameters. We rarely excluded sample preparations from the analysis, if the virus particles constantly revealed structural defects, such as particle deformation or impaired visibility of ultrastructural detail, which indicate inefficient preservation by chemical fixation and follow-up sample preparation.

The magnification setting of the microscope was monitored on a regular basis using the MAG*I*CAL calibration reference standard and did not change with time. We calculated a correction factor for the constant deviation, which was small and therefore not included in the standard calibration table of the imaging system. All values shown in the publication represent corrected values. The values of the individual particle measurements presented in the measurement file provided with the images in the datasets are un-corrected values, if not stated otherwise. The original pixel size of the images was 0.16675 nm and was corrected to 0.1641 nm in the image files.

## Usage Notes

The images may be used for developing automated image analysis tools for extracting morphometric data of virus particle populations which is required in studying the reasons for an altered infectivity of virus variants^31^. The images can be used to build machine-learning workflows which require suitable reference material for their training. The datasets published along with our previous publication^3^ were used to develop blind zero-shot algorithms for microscopy image denoising^32^ which demonstrates the general usefulness of such systematically generated image datasets. Moreover, in-depth analyses of more SARS-CoV-2 variants can extend our datasets and capture possible trends in virus morphometry during further evolution of the virus which would support understanding the epidemiology of the different variants.

## Data Availability

No customized code was used for generating the data.
